# A prospective registry analysis of psychosocial and metabolic health between women with and without metabolic syndrome after a complicated pregnancy

**DOI:** 10.1186/s12905-022-02035-y

**Published:** 2022-11-21

**Authors:** Emily Aldridge, K. Oliver Schubert, Maleesa Pathirana, Susan Sierp, Shalem Y. Leemaqz, Claire T. Roberts, Gustaaf A. Dekker, Margaret A. Arstall

**Affiliations:** 1 Adelaide Medical School, University of Adelaide, Adelaide, South Australia Australia; 2 Robinson Research Institute, University of Adelaide, Adelaide, South Australia Australia; 3 Department of Cardiology, Northern Adelaide Local Health Network, Elizabeth Vale, South Australia Australia; 4 Division of Mental Health, Northern Adelaide Local Health Network, Elizabeth Vale, South Australia Australia; 5 Headspace Early Psychosis, Sonder, Adelaide, South Australia Australia; 6 Flinders Health and Medical Research Institute, Flinders University, Bedford Park, South Australia Australia; 7 Department of Obstetrics & Gynaecology, Northern Adelaide Local Health Network, Elizabeth Vale, South Australia Australia

**Keywords:** Maternal health, Metabolic syndrome, Pregnancy complications, Maternal mental health

## Abstract

**Purpose:**

Pregnancy complications affect over one quarter of Australian pregnancies, and this group of mothers is vulnerable and more likely to experience adverse cardiometabolic health outcomes in the postpartum period. Metabolic syndrome is common in this population and may be associated with postpartum mental health issues. However, this relationship remains poorly understood. To compare the differences in psychosocial parameters and mental health outcomes between women with metabolic syndrome and women without metabolic syndrome 6 months after a complicated pregnancy.

**Methods:**

This study is prospective registry analysis of women attending a postpartum healthy lifestyle clinic 6 months following a complicated pregnancy. Mental health measures included 9-item Patient Health Questionnaire (PHQ-9), 7-item Generalised Anxiety Disorder questionnaire (GAD-7), self-reported diagnosed history of depression, anxiety and/or other psychiatric condition, and current psychotropic medication use.

**Results:**

Women with metabolic syndrome reported significantly more subjective mental health concerns, were more likely to have a history of depression and other psychiatric diagnoses and were more likely prescribed psychotropic medications. However, there were no significant differences in PHQ-9 and GAD-7 scores.

**Conclusion:**

Amongst new mothers who experienced complications of pregnancy, those with metabolic syndrome represent a particularly vulnerable group with regards to psychosocial disadvantage and mental health outcomes. These vulnerabilities may not be apparent when using common standardised cross-sectional mental health screening tools such as PHQ-9 and GAD-7.

**Supplementary Information:**

The online version contains supplementary material available at 10.1186/s12905-022-02035-y.

## Introduction

Maternal complications of pregnancy, including hypertensive disorders of pregnancy, gestational diabetes mellitus, intrauterine growth restriction, spontaneous preterm birth, and placental abruption, affect over 30% of Australian pregnancies and are associated with an increased risk of future cardiometabolic disease [1–7]. In recent years, attention has turned to preventing chronic disease in these women who are at higher risk of adverse future cardiovascular health, through lifestyle and behaviour change interventions. The first nurse-led intervention clinic for this purpose was introduced in 2018 in South Australia [8]. Traditional cardiovascular risk calculators are generally unsuitable for determining cardiovascular risk in young and female cohorts [9]. Metabolic syndrome, which refers to a cluster of the most dangerous heart attack risk factors [10], is a useful alternative for assessing cardiovascular risk in young women.

With similar incidence, peripartum mental health issues are common in Australia, with depression or anxiety affecting approximately 1 in 5 mothers of children under the age of 2 years [11]. There is some literature to suggest that adverse maternal mental health is associated with complications of pregnancy. When controlling for age, ethnicity and pre-pregnancy body mass index (BMI), maternal mood and anxiety disorders were found to be associated with a two-fold increased risk of preeclampsia [12]. Women diagnosed with gestational diabetes report more depressive symptoms both antenatally and postpartum [13, 14]. Maternal self-reported anxiety and depression during pregnancy have been found to be associated with poor birth outcomes including preterm birth and low birth weight [15], which was further confirmed in a recent systematic review [16]. Higher levels of maternal stress, anxiety and depression measured at 20 weeks gestation have also been found to be associated with fetal growth restriction, and especially for male infants [17]. The mechanisms of the relationship between maternal depression and anxiety and complications of pregnancy remain unclear, as does the extent (if any) to which perinatal mental health contributes to the increase in lifetime cardiometabolic risk.

Psychiatric conditions are associated with an increased risk of metabolic syndrome [18], and depression, in particular, has been shown to have a bidirectional relationship with metabolic syndrome [19, 20]. This relationship is further mediated by socioeconomic status, with a recent study finding that lower socioeconomic status was associated with a more adverse metabolic health profile and a higher prevalence of depression [21]. It has been proposed that obesity and inflammation, in addition to hypothalamic pituitary adrenal axis and sympathetic nervous system activation, may influence both depression and metabolic syndrome, and therefore that these same mechanisms may underlie pregnancy complications and adverse peripartum mental health [22]. However, this hypothesis remains untested, and the area is under researched. Furthermore, maternal follow-up after pregnancy usually ceases 6–12 weeks postpartum, and as such little information is routinely collected on the metabolic and mental health of mothers after the immediate postpartum period.

This study aims to explore the relationship between metabolic syndrome, psychosocial characteristics, and mental health in a selected cohort of highly vulnerable mothers at 6 months postpartum. It is hypothesised that women with metabolic syndrome will have more psychosocial problems and poorer mental health than women without metabolic syndrome.

## Methods

### Study design and setting

This was a prospective registry analysis of the postpartum lifestyle intervention clinic at the Lyell McEwin Hospital (LMH) [8]. The LMH is a public tertiary acute-care facility providing obstetric care, adult cardiac and intensive care services, and neonatal care for infants for ≥32 weeks’ gestation located within the Northern Adelaide Local Health Network (NALHN), South Australia. The NALHN area is characterised by a population with low socioeconomic status with high rates of CVD morbidity and mortality, and is among Australia’s most disadvantaged suburban communities [23]. The Central Adelaide Local Health Network Human Research Ethics Committee approved the study and waived the requirement for informed written consent [HREC/16/TQEH/258].

### The postpartum lifestyle intervention clinic

Methods for the hospital-based outpatient clinical service and associated quality assurance registry have been previously described [8]. Briefly, to be eligible for referral to the postpartum intervention clinic, women must have experienced at least one of the following complications in their index pregnancy: hypertensive disorder of pregnancy requiring medical therapy or resulting in birth < 37 weeks’ gestation, gestational diabetes mellitus requiring metformin or insulin therapy, spontaneous preterm birth < 34 weeks’ gestation, intrauterine growth restriction, delivery of a small for gestational age infant at <5th customised birth centile, or placental abruption. At approximately 6 months postpartum, participants underwent a thorough health assessment and received individualised health counselling from a nurse practitioner (S.S.) expert in cardiovascular prevention and education. This included suggested dietary and exercise improvements, as well as any additional testing and referrals to specialists where appropriate. Variables were collected from a combination of patient self-report and abstraction from the hospital medical record. Information collated and included in the registry included patient demographics, medical history, family history, current medications, alcohol, drug and smoking practices, obstetric history, cardiovascular and metabolic screening pathology results, systolic and diastolic blood pressures, height, weight, and waist circumference. Blood pressure was measured using an oscillometric pulse wave analysis device, the USCOM BP+ [USCOM, Sydney, Australia]. Current depression and anxiety symptom screening was performed using the 9-item Patient Health Questionnaire (PHQ-9) and the 7-item General Anxiety Disorders questionnaire (GAD-7). To determine prevalence of psychiatric conditions, participants were asked if they had a current or previous history of diagnosed depression, anxiety, or other psychiatric condition, which was recorded and dichotomised as either ‘Yes’ or ‘No’. These questionnaires are validated self-report questionnaires that assess symptoms over the preceding fortnight [24, 25]. Social support was assessed using the Medical Outcomes Study Social Support Scale (MOS-SSS), a validated self-report frequency scale that sub-groups support into four categories; emotional/informational support, tangible support, affectionate support, and positive social interaction [26].

### Study inclusion / exclusion criteria

Women who attended the postpartum clinic from 7th August 2018 to 31st October 2021 were included. Those who were pregnant at the time of appointment or failed to complete the pathology testing were excluded as metabolic syndrome status could not be accurately determined.

### Metabolic syndrome definition

Metabolic syndrome was defined as the presence of any three of the following five risk factors [10]:

- Elevated waist circumference with ethnicity specific values defined by the International Diabetes Federation [27], which for women is ≥80 cm for all ethnicities
- Elevated triglycerides of ≥1.7 mmol/L, or drug treatment for this lipid abnormality
- Reduced HDL cholesterol of < 1.3 mmol/L, or drug treatment for this lipid abnormality
- Elevated systolic blood pressure of ≥130 mmHg and/or diastolic blood pressure of ≥85 mmHg, or antihypertensive drug treatment
- Elevated fasting glucose of ≥5.6 mmol/L, or drug treatment of elevated glucose.

### Outcomes

The primary outcomes of interest for this study included depression and anxiety screening scores as assessed by the PHQ-9 and the GAD-7, as well as the prevalence of self-reported history or current experience of depression, anxiety or other psychiatric condition, and the prevalence of current psychotropic medication use.

Secondary outcomes included social support scores and psychosocial risk factors.

Other reported outcomes in this study included individual cardiovascular and metabolic risk factors, such as waist circumference, body mass index, peripheral systolic and diastolic blood pressures, triglycerides, HDL cholesterol, fasting plasma glucose, fasting insulin, and homeostasis model assessment-estimated insulin resistance (HOMA-IR).

All study measures were collected and current at the 6 months postpartum timepoint.

### Analysis

Continuous variables are presented as mean and standard deviation for normally distributed data, or median and interquartile range for non-normally distributed data. Categorical data are presented as count and percentage. Values were rounded to the nearest two decimal places. Chi-square, and Fisher’s exact tests where appropriate, were used to compare the difference in proportions between groups for categorical variables. Independent samples t-tests, or Mann Whitney U-tests where data were not normally distributed, were used to assess the difference in mean values between groups for continuous variables. Multiple linear regressions were performed to assess the influence of metabolic syndrome and other co-variates on the primary outcome variables. The PHQ-9 and GAD-7 variables were log transformed to approximate normality, and results are reported as ratio of geometric means of the scores in women who had metabolic syndrome compared to those without, and the corresponding 95% confidence intervals. A two-sided *p*-value of < 0.05 was deemed statistically significant. All analyses were conducted using IBM SPSS Statistics for Windows, version 28.0 (Armonk, NY: IBM Corp).

## Results

A total of 312 participants were included in the registry during the study time period. Five participants were excluded due to being pregnant again at the time of the appointment, and an additional fifteen were excluded due to not completing the pathology tests required to determine metabolic syndrome status. This resulted in a final sample size of 292 for the present analysis.

A total of 106 participants (36.3%) met the criteria for metabolic syndrome. Participant demographics were stratified by group and are presented in Table 1. Participants with metabolic syndrome were less likely to be university educated (*p* < 0.05) or employed (*p* < 0.001) compared to those without metabolic syndrome.

**Table 1:** Participant demographics at six months postpartum

	Metabolic syndrome, ***n*** = 106 Mean ± SD, median [interquartile range], or n (%)	No metabolic syndrome, ***n*** = 186 Mean ± SD, median [interquartile range], or n (%)	***p***-value
Time to follow up, months	6.0 [6–8]	6.0 [6–7]	0.02^a^
Age	32.61 ± 5.12	31.98 ± 5.24	0.32
BMI, m/kg^2^	35.76 ± 7.94	29.24 ± 6.96	< 0.001^a^
Gravidity	2.89 ± 2.06	2.70 ± 1.61	0.39
Parity	2.18 ± 1.58	2.02 ± 1.13	0.37
Australian born	59 (55.66)	102 (54.84)	0.89
Interpreter required	16 (15.09)	29 (15.59)	0.91
Ethnicity			0.92
*Caucasian*	59 (55.66)	100 (53.76)	
*Aboriginal*	2 (1.89)	4 (2.15)	
*African*	3 (2.83)	12 (6.45)	
*Asian*	21 (19.81)	37 (19.89)	
*Hispanic*	1 (0.94)	2 (1.08)	
*Indian subcontinent*	10 (9.43)	15 (8.06)	
*Middle eastern*	10 (9.43)	16 (8.60)	
Referring complication^b^			
HDP, *n* = 104	42 (39.62)	62 (33.33)	0.28
GDM, *n* = 206	82 (77.36)	124 (66.66)	0.05
IUGR, *n* = 25	6 (5.66)	19 (10.22)	0.18
SPTB, *n* = 6	3 (2.83)	3 (1.61)	0.48
PA, *n* = 4	1 (0.94)	4 (2.15)	0.64

*Abbreviations*: *SD* Standard deviation, *BMI* Body mass index, *HDP* Hypertensive disorders of pregnancy, *GDM* Gestational diabetes mellitus, *IUGR* Intrauterine growth restriction, *SPTB* Spontaneous preterm birth, *PA* Placental abruption

^a^Denotes statistical significance

^b^46 participants had more than one referring complication

### Psychosocial risk factors

Adverse social and mental health risk factors in women with and without metabolic syndrome at 6 months postpartum are shown in Table 2. There were no statistically significant differences in any of the social risk factors, but there was a higher percentage of reported current or previous diagnosed depression (35.8% vs 24.7%), other current or previous psychiatric conditions (16.0% vs 4.3%) and current psychotropic medication use (16.9% vs. 8.6%) in the metabolic syndrome group compared to the group without metabolic syndrome. There was no difference in the percentage of self-reported anxiety.

**Table 2:** Psychosocial risk factors according to metabolic syndrome status, *n* = 292

Adverse social and mental health factors	Metabolic syndrome, ***n*** = 106 n (%)	No metabolic syndrome, ***n*** = 186 n (%)	***p***-value
University educated^b^	19 (20.43)	59 (34.3)	0.02^a^
Currently employed	43 (40.57)	112 (60.2)	0.001^a^
SEIFA IRSAD	931.50 [868–965]	936.00 [868–964]	0.48
Trauma or grief	4 (3.7)	5 (2.7)	0.61
Relationship issues	6 (5.6)	8 (4.3)	0.60
Homelessness or housing issues	0	1 (0.5)	0.45
Sexual abuse	1 (0.9)	2 (1.1)	1.00
Emotional abuse	2 (1.8)	2 (1.1)	0.57
Domestic violence	1 (0.9)	3 (1.6)	1.00
Current psychotropic medication	18 (16.9)	16 (8.6)	0.03^a^
Depression^c^ *“Do you have a history of or current diagnosis of depression?”*	38 (35.8)	46 (24.7)	0.04^a^
Anxiety^c^ *“Do you have a history of or current diagnosis of anxiety?”*	34 (32.1)	59 (31.7)	0.95
Other diagnosed psychiatric condition *“Do you have a history of or current diagnosis of any other mental illness?”*	17 (16.0)	8 (4.3)	0.001^a^
Any psychiatric condition	52 (49.1)	74 (39.8)	0.12

*Abbreviations*: *SEIFA IRSAD* Socioeconomic Index for Areas Index of Relative Socioeconomic Advantage and Disadvantage

^a^Denotes statistical significance

^b^Data missing for 27 participants: 13 from metabolic syndrome group, 14 from control group

^c^Includes current and/or history of depression and anxiety treated with medication or psychotherapy

### Mental health screening scores

Differences between PHQ-9, GAD-7 and MOS-SSS scores according to metabolic syndrome status are shown in Table 3. The cardio-metabolic risk factors according to metabolic syndrome status are presented in Table 4. As expected, there were statistically significant differences for every cardio-metabolic risk factor between groups.

**Table 3:** Depression and anxiety symptoms and social support scores according to metabolic syndrome status

	Metabolic syndrome Median [interquartile range]	n	No metabolic syndrome Median [interquartile range]	n	***p***-value
PHQ-9 Score	4 [2–8]	103	4 [1–8]	179	0.63
GAD-7 Score	3 [0–8]	102	3 [1–8]	178	0.40
MOS-SSS scale	87.40 [69.41–96.93]	88	87.92 [72.61–98.13]	161	0.72
MOS-SSS score	4.50 [3.78–4.88]	88	4.52 [3.90–4.93]	161	0.72
Emotional/informational support	4.13 [3.63–5.0]	87	4.25 [3.63–5.0]	160	0.64
Tangible support	4.25 [3.25–5]	88	4.25 [3.5–5]	160	0.62
Affectionate support	5.00 [4.33–5]	88	5.00 [4.33–5]	159	0.69
Positive social interaction	4.33 [3.67–5.0]	88	4.33 [3.67–5.0]	161	0.85

*Abbreviations*: *PHQ-9* Patient Health Questionnaire 9-item, *GAD-7* General Anxiety Disorders 7-item questionnaire, *MOS-SSS* Medical Outcomes Study Social Support Survey

**Table 4:** Cardio-metabolic risk factors according to metabolic syndrome status, *n* = 292

	Metabolic syndrome, ***n*** = 106 Median [interquartile range] or mean ± SD	No metabolic syndrome, ***n*** = 186 Median [interquartile range] or mean ± SD
Waist circumference, cm	105.95 [93.2–116.7]	91.50 [82.8–100.0]
Peripheral SBP, mmHg	128 [119–136]	119 [111–127]
Peripheral DBP, mmHg	79 ± 10	72 ± 10
^a^Triglycerides, mmol/L	2.00 [1.2–2.5]	0.80 [0.6–1.2]
^a^HDL cholesterol, mmol/L	1.10 [1.0–1.2]	1.40 [1.3–1.6]
^a^Glucose, mmol/L	5.20 [5.0–5.9]	4.80 [4.5–5.1]
^a^Insulin, mU/L	19.80 [13.0–29.3]	8.70 [6.0–13.4]
HOMA-IR index	4.49 [2.8–6.9]	1.93 [1.2–2.9]

*Abbreviations*: *SD* Standard deviation, *SBP* Systolic blood pressure, *DBP* Diastolic blood pressure, *HDL* High-density lipoprotein, *HOMA-IR* Homeostasis model assessment-estimated insulin resistance

^a^Fasting

The PHQ-9 and GAD-7 scores were dichotomised as either ‘score of ≥10’ (which denotes ‘likely major depressive disorder’ and ‘likely generalised anxiety disorder’ for PHQ-9 and GAD-7, respectively) and ‘score of <10’). Chi-square tests were performed to compare the difference in proportions of scores between participants with metabolic syndrome and those without metabolic syndrome. For the PHQ-9, 20.4% of participants with metabolic syndrome scored 10 or above (likely major depressive disorder) compared to 19.0% without metabolic syndrome (*p* = 0.77). On the GAD-7, 18.6% of participants with metabolic syndrome scored ≥10 or ‘likely generalised anxiety disorder’ compared to 19.1% of patients without metabolic syndrome on the GAD-7 (*p* = 0.92).

Multiple linear regressions were performed to estimate the difference in PHQ-9 and GAD-7 scores between participants with metabolic syndrome and those without, adjusting for their employment status, education level, current psychotropic medication use, current or history of depression, current or history of anxiety, and current or history of other psychiatric condition (Supplementary Tables 1 and 2). Participants with metabolic syndrome had 17.2% lower score (ratio of geometric means 0.83 (95% CI 0.65, 1.05)) for PHQ-9 on average than those without metabolic syndrome, although this was not statistically significant. Participants with metabolic syndrome had 31.4% lower score (ratio of geometric means 0.69 (95% CI 0.52, 0.90)) for GAD-7 on average than those without metabolic syndrome, and this was statistically significant (*p* < 0.01).

Pearson’s correlations were used to assess the correlations between PHQ-9 and GAD-7 scores with the metabolic syndrome biomarkers of glucose, triglycerides, HDL cholesterol, and insulin. None of these variables were strongly correlated nor statistically significant.

## Discussion

The aim of this study was to explore the relationships between metabolic syndrome, psychosocial characteristics, and mental health outcomes in a cohort of women who gave birth following a complicated pregnancy 6 months earlier. It was hypothesised that women with metabolic syndrome would have overall poorer mental health outcomes compared to women who did not have metabolic syndrome.

Although the number of women who met the criteria for ‘likely major depressive disorder’ on the PHQ-9 and GAD-7 did not differ significantly between groups, there was a significantly higher percentage of adverse psychosocial factors in the metabolic syndrome group, including self-reported diagnoses of depression or other psychiatric disorders, or current psychotropic medication use. These findings reflect the psychosocial vulnerability of women with metabolic syndrome compared to those without metabolic syndrome. There are a number of possible explanations why these psychosocial differences were not detectable when applying the screening tools. Firstly, although more psychosocially vulnerable, women with metabolic syndrome may not have been necessarily suffering from a diagnosable major depressive episode at the time of assessment. It is also possible that other mental health difficulties, aside from major depressive disorder or generalised anxiety disorder, were present in this group but not detected as the appropriate screening tools were not used. Although the PHQ-9 and GAD-7 are well validated tools, they are still brief self-report measurements, and more sophisticated assessments may have yielded different results. Secondly, approximately one-fifth of women in both groups scored ≥10 on either PHQ-9 or GAD-7, indicating ‘likely major depressive disorder’ or ‘likely generalised anxiety disorder’, respectively. This is a higher percentage than we may typically expect to see in a relatively early postnatal population and is certainly higher than rates reported in the literature. For example, a 2018 Australian study of antenatal and postnatal depression reported rates of antenatal and postnatal depression at 6.2 and 3.3%, respectively [28]. The overall high number of women scoring ≥10 on either screening tool in our study may have obscured the true differences between the groups. There may also have been additional confounding factors that were not controlled for in the linear regression analysis. Finally, the higher rates of psychotropic medication use in the metabolic syndrome group may be associated with effective management of depression and anxiety symptoms, thus reducing cross-sectionally assessed PHQ-9 and GAD-7 scores. Important to note, however, is that some psychotropic medications are associated with weight gain which may initiate or exacerbate metabolic syndrome [29]. Use of some antipsychotics during pregnancy have also been shown to increase the risk of some complications of pregnancy, such as gestational diabetes and preterm birth [30]. This could be an alternative explanation for the higher rates of psychotropic medication use in the metabolic syndrome group.

Metabolic syndrome has recently been associated with higher PHQ-9 scores in general adult populations [31, 32], although this association was not confirmed in our study of relatively young women. It is also possible that, due to the self-report nature of the screening tools used in this study, some women did not accurately report their depressive symptoms on the screening tools which were administered at the beginning of the appointment. Fear and anxiety of potential consequences play an important role in influencing a person’s willingness to discuss their mental health [33]. Previous findings in the primary care setting have found that over half of patients are reluctant to honestly disclose their mental health concerns to their physician due to fear of being prescribed unwanted antidepressants or other course of psychiatric treatment, privacy concerns, or due to anxiety of being considered as ‘a psychiatric patient’ [34]. Levels of stigma may even be increased in socioeconomically disadvantaged populations and in cohorts of new mothers, who may fear negative repercussions such as separation from their baby and family. This is likely to be the case at least in mothers with severe psychiatric illness and those with a significant history of engagement with the mental health system [35]. Finally, some women may worry about the perception of feeling depressed recently after having a baby. Despite the growing awareness and decreasing stigma surrounding peripartum depression, a 2019 meta-synthesis of qualitative studies focusing on marginalised women with postpartum depression found that many women reported feeling ‘as though something was wrong’ with them [36]. Guilt, avoidance, detachment, and social comparison also seem to be important feelings associated with postpartum distress [37]. Improving relationships and communication between healthcare practitioners and women may assist to build trust and reduce barriers to disclosing and appropriately treating mental health concerns [38]. The postpartum clinic appointments usually last for 45–60 minutes; it is possible that over the course of the appointment, the women felt more comfortable to report mental health concerns, which could account for the disparity between the screening tool results obtained and the depression and anxiety rates reported from the global questions.

Significantly more women without metabolic syndrome were employed and university educated, compared to the women with metabolic syndrome. This is consistent with previous literature that has reported a relationship between socioeconomic disadvantage (determined using one or a combination of level of education, income, and employment) and a higher incidence of metabolic syndrome, especially for women [39–43]. Obesity and socioeconomic disadvantage have both been found to be independently associated with a higher risk of antenatal depression in a cohort of pregnant primiparous women [44]. In another Australian study, socioeconomic disadvantage and being from a culturally and linguistically diverse population were risk factors for both antenatal and postnatal depression [28]. Although there were no differences in socioeconomic status as measured by SEIFA-IRSAD in our study, our cohort was recruited from a socioeconomically disadvantaged population.

An important finding of our study was that over 20% of the entire cohort reported mental health concerns at the time of their 6-month postpartum clinic appointment. Peripartum mental health services typically cease in South Australia by 12 weeks postpartum, and women then need to rely on their general practitioners as the first port of call for mental health services. In a recent study of maternal mental health in the United States, 31% of women were initially referred for mental healthcare after 8 weeks postpartum, when typical obstetric support has ended [45]. Our findings confirm the desirability for additional routine postpartum screening to occur later than 6–12 weeks postpartum. This would be a significant challenge to implement due to the overburdened mental health system in Australia and those around the world.

Metabolic syndrome is associated with an increased prevalence of depression [19], regardless of age, sex, BMI, smoking status, socioeconomic status, and lifestyle factors [46]. The association between anxiety and metabolic syndrome has been more contentious, but a 2017 meta-analysis reported a significant positive association between the two [47]. There has been far less attention paid to metabolic syndrome in young mothers, let alone how depression and anxiety may influence their cardiometabolic health. Future studies should focus further on both postpartum mental health and cardiometabolic risk.

It remains difficult to characterise the relationship between cardiometabolic risk, depression and other mood disorders, and complications of pregnancy. A significant number of potential pleiotropic genes and biological pathways are likely to be shared between both cardiometabolic diseases and mood disorders, including depression and bipolar disorder [48]. The higher incidence of complications of pregnancy in women with typical cardiovascular risk factors, such as smoking [49], obesity and pre-existing hypertension [50–52], are likely to, at least in part, be influenced by genetic predispositions that may in turn also predispose to mood disorders. Further exploration of the genetic overlap between cardiometabolic traits and mood disorders may also lead to improved preventative strategies for both chronic conditions [53].

A recent study of depression profilers and immuno-metabolic dysregulation found ‘atypical, energy-related symptoms’ of depression (characterised by increased appetite, increased weight, hypersomnia, leaden paralysis, and low energy) were associated with poorer inflammatory and metabolic health, both cross-sectionally and longitudinally [54]. Interestingly, the ‘anxious stress symptoms’ of depression (characterised by feeling tense, restlessness, concentration/worrying, fear of awful events, feeling like losing control) were not strongly associated with inflammation or metabolic health [54]. Further research into the mechanisms and genetic influences of cardiometabolic health, and how these may overlap with mental and pregnancy health, is needed.

## Limitations

This study has a number of limitations warranting discussion. Firstly, no objective data on antenatal or pre-pregnancy mental or metabolic health status were collected so baseline mental health and metabolic syndrome status prior to attending the postpartum clinic are unknown. Secondly, gestational diabetes is strongly linked with metabolic syndrome [55], and the percentage of gestational diabetes was higher in the group of women with metabolic syndrome, although this did not quite reach statistical significance. The previous diagnoses of depression, anxiety or other mental health conditions relied on patient self-report only, with a minority of cases able to be confirmed through evidence in the medical case notes. There were also some missing data from the PHQ-9 and GAD-7 scores due to inadequate completion, patient refusal to complete, or lack of time during the appointment. Finally, the sample size may have limited our ability to adequately investigate the potential role of confounders on depression and anxiety symptom scores.

## Conclusion

The results of this study highlight the need for ongoing research of the relationship between cardiometabolic and mental health in women who have recently experienced a complicated pregnancy. Pregnancy and the early postpartum period remain an opportune time to engage with women at risk of chronic disease due to their increased contact with the healthcare system and motivation to make lifestyle changes for themselves and the benefit of their baby. Ongoing mental health screening for new mothers may be extremely beneficial to their mental and emotional wellbeing, something that should be explored with further research. This in turn may also have potential benefits for their ongoing cardiometabolic health.

## Supplementary Information

**Additional file 1: Supplementary Table 1.** Multiple linear regression model estimating difference in PHQ-9 scores between metabolic syndrome status. **Supplementary Table 2.** Multiple linear regression model estimating difference in GAD-7 scores between metabolic syndrome status.

## Data Availability

The datasets generated and/or analysed during the current study are not publicly available due to local ethical and legal requirements but are available from the corresponding author on reasonable request.
