# Supplementary material for: A prospective registry analysis of psychosocial and metabolic health between women with and without metabolic syndrome after a complicated pregnancy
Source: BMC Womens Health. 2022 Nov 21;22:461. doi: 10.1186/s12905-022-02035-y (PMC9677702; doi:10.1186/s12905-022-02035-y)
Supplement: Supplementary file 1 — Additional file 1: Supplementary Table 1. Multiple linear regression model estimating difference in PHQ-9 scores between metabolic syndrome status. Supplementary Table 2. Multiple linear regression model estimating difference in GAD-7 scores between metabolic syndrome status. [file 12905_2022_2035_MOESM1_ESM.docx]

Supplementary Table 1. Multiple linear regression model estimating difference in PHQ-9 scores between metabolic syndrome status

| **Variable** | **Ratio of geometric means (95% CI)** |
| --- | --- |
| Metabolic syndrome | 0.83 (0.65, 1.05) |
| Employment status | 0.91 (0.72, 1.14) |
| Education level | 1.10 (0.86, 1.42) |
| Current psychotropic medication use | 1.07 (0.73, 1.56) |
| Current or history of depression | 1.86 (1.39, 2.49) |
| Current or history of anxiety | 1.50 (1.14, 1.97) |
| Current or history of other psychiatric condition | 2.11 (1.37, 3.23) |

Supplementary Table 2. Multiple linear regression model estimating difference in GAD-7 scores between metabolic syndrome status

| **Variable** | **Ratio of geometric means (95% CI)** |
| --- | --- |
| Metabolic syndrome | 0.69 (0.52, 0.90) |
| Employment status | 0.81 (0.90, 1.57) |
| Education level | 1.19 (1.22, 3.19) |
| Current psychotropic medication use | 1.11 (0.72, 1.70) |
| Current or history of depression | 2.09 (1.51, 2.90) |
| Current or history of anxiety | 1.81 (1.33, 2.45) |
| Current or history of other psychiatric condition | 1.98 (1.22, 3.19) |
